# Reproductive health services utilization and its associated factors among adolescents in Debre Berhan town, Central Ethiopia: a community-based cross-sectional study

**DOI:** 10.1186/s12978-018-0659-4

**Published:** 2018-12-27

**Authors:** Kenean Getaneh Tlaye, Mesfin Abebe Belete, Tefera Mulugeta Demelew, Mikiyas Amare Getu, Feleke Hailemichael Astawesegn

**Affiliations:** 1 Nursing Department, Health Science Faculty, Woldia University, Woldia, Ethiopia; 2 School of Nursing and Midwifery, College of Health Science, Addis Ababa University, Addis Ababa, Ethiopia; 3 School of Public Health, College of Health Sciences and Medicine, Hawassa University, Hawassa, Ethiopia

**Keywords:** Reproductive health, Adolescents, Service utilization

## Abstract

**Background:**

Utilization of reproductive health services is an important component in preventing adolescents from different sexual and reproductive health problems. As a result, the extent of their service utilization should be determined before implementing any kind of interventions. Therefore, this study was aimed at assessing the level of reproductive health services utilization and its associated factors among adolescents who live in Debre Berhan town.

**Methods:**

A community-based cross-sectional method was employed in this study from April 5–May 1, 2016. A multi-stage systematic sampling technique was applied to select a total of 648 adolescents living in 5 randomly selected kebeles of Debre Berhan town. Moreover, a logistic regression was done to identify independent predictors of reproductive health service utilization.

**Results:**

Accordingly, the major findings of this study reveals that about one-third (33.8%) of adolescents utilized at least one of reproductive health services. Adolescents who had discussed sexual and reproductive health issues with their sexual partner and peers were two times more likely to use reproductive health services than their counter parts (AOR = 2.368, 95% CI: 1.168–4.802 and AOR = 2.360, 95% CI: 1.155–4.820 respectively). Adolescents who weren’t co-resided with both their parents were also about two times more likely to utilize reproductive health service than those who were living together (AOR = 2.570, 95% CI = 1.155–4.820). Positive perception of oneself towards acquisition of Human Immunodeficiency Virus urged the adolescents to use RH services twice than those who didn’t perceive themselves as risky (AOR = 2.231, 95%CI: 1.001–4.975).

**Conclusion:**

Succinctly speaking, the analysis of the major finding suggests that the utilization of reproductive health services among adolescents in the study area was low. Discussion with sexual partner and peers, risk perception of oneself towards the acquisition of human immune-deficiency virus was among the predictors of reproductive health services usage.

**Electronic supplementary material:**

The online version of this article (10.1186/s12978-018-0659-4) contains supplementary material, which is available to authorized users.

## Plain English summary

Reproductive Health Service includes access to information and services on prevention, diagnosis, counseling, treatment and care, and requires that all people can safely reach services without travelling a long distance or wasting time. This means services and treatments must be affordable to people based on the principle of equity. The rationale behind the study was to investigate the level of reproductive health services utilization and to find out the associated factors among adolescents in order to provide evidence-based information and recommendations to alter possible future interventions.

A community based cross-sectional study design was conducted on selected adolescents from 5 ‘kebeles’ of Debre Berhan town. In doing so, Adolescents who have sought or received at least one service out of the four identified RH services under the study, that is, Sexual and Reproductive Health (SRH) information and education service; modern contraceptive service; Voluntary Counseling and Testing for HIV (VCT) service; and Sexually Transmitted Infections (STI) diagnosis and treatment service utilization were considered to be used by the adolescents.

Six hundred forty-eight participants responded to the questionnaires yielded a response rate of 95%. The mean age of the respondents was 17.13 (SD + 1.33). Respondents who had good knowledge were 356 (54.9%), and 68.5% of all adolescents had high geographical accessibility to reproductive health service. Adolescents who had discussed sexual and reproductive health issues with their sexual partner and peers, adolescents who didn’t resided with both their parents and positive perception of oneself towards acquisition of HIV/AIDS urged adolescents to use those services.

In conclusion; despite the major proportion of adolescents having good knowledge and high geographical accessibility to reproductive health services, the use of RH services in the study area was low.

## Background

According to World Health Organization (WHO), adolescents are people between 10 and 19 years of age; they make 20% of the world’s population, of whom 85% live in developing countries. Adolescence is characterized by significant physiological, psychological, and social changes that put them on high risk of sexual and reproductive health related problems. This is because adolescents were considered to be relatively healthy, citizens who lives without a heavy “burden of disease” [1, 2].

Reproductive health care is defined as the constellation of methods, techniques, and services that contribute to reproductive health and well-being by preventing and solving sexual health problems [3].

The WHO stated that about one half of all HIV infections worldwide occur among people aged 25 years and under 25 years of age. In addition, up to 100 million youths become infected with curable sexually transmitted diseases. Every year an estimated of 1.7 million youths lose their life prematurely due to preventable or treatable problems such as accidents, violence, pregnancy related complications, and other illnesses. For instance, in Africa, it was estimated that 60% of all new HIV infections occur among the youth aged 15–19 years [2]. Sub-Saharan Africa remains the most affected region in the world with an estimate of 22.5 million people living with HIV and approximately 1.7 million new infections occurred in the region. Furthermore, premarital sexual activity has the highest rate in Sub-Saharan Africa, where more than half of girls aged 15–19 have sexual experience [4].

According to the Ethiopian Demographic and Health Survey (EDHS) of 2011, among the age group of 15–24 years, HIV prevalence was 0.4%. That is, the utilization of family planning services in the existing health care system by young people was also very low. As a result, there is a high rate of unwanted pregnancies which often result in abortions and its complications. From all posts, among incomplete abortion treatment seekers, the majority (67.2%) of them were under 24 years of age. In addition, the 2011 EDHS figured out that contraceptive use among adolescents were lower when compared with other age groups [5].

The concern about adolescent sexual and reproductive health has grown due to unprecedented increasing rates of sexual activity, early pregnancies and sexually transmitted infections including human immune deficiency virus among adolescents [6, 7], which in turn negatively affect their health, productivity, and quality of life.

As a result, the Federal Ministry of Health (FMOH) launched several strategies to promote adolescents and youth reproductive health services such as the National Reproductive Health Strategy implemented during 2006–2015, and National Adolescent and Youth Reproductive Health Strategy implemented during 2007–2015. Similarly, Standards on Youth Friendly Reproductive Health Services; and also tools for planning, implementation, and monitoring at different levels of the health system were prepared. What is more, youth friendly health services were established so as to provide RH services to adolescents and youths in line with the existing health facilities. In spite of all the above facts, Ethiopia still has huge gap in accessing adolescents and youth with reproductive health services [8].

The evidences discussed above imply that adolescents’ health problems were related to their utilization of RH service either directly or indirectly because it is obvious that the more readily the services are utilized, the less the problems will be. Even though certain factors were recognized as reasons for the low-level RH service utilization so far, additional in-depth investigation is needed especially at areas where no prior studies have been conducted. Therefore, the rationale behind this research was to investigate the level of reproductive health services utilization and to find out associated factors among adolescents who live in Debre Berhan town, in order to provide evidence-based information and recommendations for possible future interventions. It also generates relevant information that could help to design appropriate RH programs for adolescents.

## Methods

### Study area and period

Cross-sectional study was conducted from April 5–May 1, 2016 in Debre Berhan town, North Shoa, Ethiopia with an objective to assess the reproductive health service utilization and to find out associated factors among adolescents of age 15–19 years. Debre Berhan town is located at 9°41’N 39°32′E/9.683°N 39.533°E, that is, 130 km North of Addis Ababa, the capital city of Ethiopia. It has 2840 m elevation above sea level and covers 14.71km^2^. The town is founded by Emperor Zara Yakob in 1456 [9], and it has 9 administrative kebeles. According to the information obtained from the District Health Office, in 2015/16 population data, the total population size of the district was 92,887, out of which 50,883 (54.78%) were females. The number of households and adolescents (10–19 years of age) in the district were estimated to be 18,453 and 22,014 respectively. In the town, there were 2 hospitals, 3 health centers, 9 health posts, 6 private clinics, and 17 pharmacies which render health services for the community. Family planning service was also available in most of the above health institutions including health posts.

### Population and sampling

#### Source of population

The source population was all households of 5 selected kebeles of Debre Berhan town that hosts adolescents of age 15–19.

#### Study population

Adolescents of age 15–19 who were living in the selected household from 5 selected kebeles of Debre Berhan town.

### Sample size and sampling procedure

#### Sample size calculation

The actual sample size was determined considering the following assumptions: level of confidence was taken to be 95% with α = 0.05 value (which yields Z α/2 = 1.96 on the standard normal distribution curve), a 5% margin of error (d = 0.05) and a proportion of 72.2% voluntary counseling and testing (VCT) service utilization obtained from previous study conducted in Gondar town (in northern Ethiopia) among adolescents age 15–19 (12). Based on this assumption, the actual sample size for the study was computed using single population proportion formula as indicated below.

$$ n=\frac{{\left(Z\frac{a}{2}\right)}^2p\left(1-p\right)}{d^2} $$

Where, n = Sample size.

z = the value of the standard normal curve score corresponding to the given confidence interval = 1.96.

p = Prevalence rate of RH service utilization = 0.72.

d = the permissible margin of error (the required precision) = 5%

$$ \frac{(1.96)^2\times 0.72\left(1-0.72\right)}{(0.05)^2}=309.8 $$

Considering Design effect of 2 and 10% non- response rate: the total sample size was

$$ 309.8\times 2+62=682 $$

### Sampling procedure

A multi-stage sampling technique was employed for the selection of the sampling units. In the district, there were 9 kebeles, of which a total of 5 kebeles were selected using lottery method. The sample size for each selected kebeles was determined proportionally to the number of households within each kebele. Then, systematic sampling method was employed to select the households where adolescents reside from each kebele assuming in a household at least one adolescent aged 15–19 will be found. In cases of household with more than one eligible respondent, only one respondent was selected by using a lottery method.

### Data collection tools and procedure

A self-administered, semi-structured questionnaire was used in the study. The questionnaire was developed by combining the John Cleland’s Illustrative questionnaire for interview-survey with young people’s [10], and also several literatures reviewed to achieve the research objectives.

The main points included in the questionnaire were: socio demographic characteristics; adolescents’ individual attribute regarding to sexuality and reproductive health; service accessibility (geographical accessibility); and four main aspects of RH services (sexual and RH information and education, modern contraceptive, voluntary counseling and testing for HIV, STI diagnosis and treatment) (See Additional file 1). The data was collected by 6 trained data collectors for 25 days under close supervision and facilitation by 1 supervisor and the principal investigator as needed**.**

### Data quality assurance

The questionnaire and consent documents were first developed in English language, and then translated into Amharic language for data collection purpose using the local language. Then finally, the subject matter experts retranslated the data back into English language in collaboration with a translation expert to check its consistency.

Face validity of the questionnaire was assessed. Items interpretability and understandability by the study participants was evaluated by pre-testing the questionnaire on 34 adolescents (5% of the total sample size) living in unselected kebeles, and the necessary correction has been taken accordingly. Six data collectors (3 females and 3 males) who were third year Nursing Department students from Debre Berhan University participated in the data collection process of the study after they were provided with the appropriate orientation and guidance about the overall project plan, and how to approach and communicate with adolescents to elicit reliable data.

The anticipated time duration to complete the questionnaire was not more than 30 min. For respondents who were absent in the first visit, a maximum of two additional schedules were established for revisit. Supervision was also done on the spot by principal investigator and supervisor. The collected data was checked for its completeness and clarity by the principal investigator daily. Data cleaning and cross-checking were also done before analysis.

### Data processing and analysis

After checking for its completeness, the data was processed and analyzed by SPSS version 21. Descriptive and analytical statistics including, bivariate and multivariate analysis was employed. Percentage, ratios, frequency distribution, measure of central tendency, and measure of dispersion have been used for describing different variables. Bivariate analysis has been used to examine association between dependent and independent variables. All variables with *p* < 0.05 in bivariate analysis was fitted in to the multiple variable logistic regression model to identify factors associated with RH service utilization. *P* value less than 0.05 was considered as a level of significance.

### Terms and operational definition

#### Adolescent

In this study adolescent stands for boys and girls between the ages of 15–19.

#### Discussion on SRH issues

Adolescents who discussed at least two SRH issues (Condom, STI/HIV/AIDS, abstinence, unwanted pregnancy, contraception) with health care provider in the last 12 months. The same was applied with peer, and sexual partner.

#### History of sexual exposure

Adolescents who ever experience sex in their life were classified as having the history of sexual exposure and not otherwise.

#### Knowledge about reproductive health service

Adolescents who score above the mean of reproductive health service related questions were labeled as having high knowledge and those score below the mean were considered as having low knowledge. The questions were about the available types of services and types of service delivery points (providers) within the area, which included a total of eight types of services and seven service delivery points.

#### Media exposure on SRH issues

Adolescents who were exposed to mass media (including radio, television, magazine/newspaper and pamphlet) on at least two SRH issues (Condom, STI/HIV/AIDS, abstinence, unwanted pregnancy, contraception) in the past 12 months.

#### Modern contraceptive service utilization

Adolescents who used any of the modern birth controlling methods (contraceptives) in the past 12 months.

#### Perception of risk towards HIV/AIDS

Adolescent’s attitude towards perceiving themselves as susceptible to HIV infection.

#### RH service accessibility

The term accessibility in this study was applied to geographical accessibility based on adolescents’ own perception. Adolescents who lived within 1.6-km (1 mile) radius distance from the nearest RH service center and from their home less than 30-min walking distance were classified as having high geographical accessibility and low otherwise.

#### RH service utilization

Adolescents having sought or received at least one of the four RH services that the study focuses on, that is, SRH information and education service, modern contraceptive service, VCT service, and STI diagnosis and treatment service utilization.

#### SRH information and education service utilization

Adolescents who received information and education regarding sexual and reproductive health issues from health worker working in any of the service providing points within the past 12 months.

#### STI diagnosis and treatment service utilization

Adolescents who ever obtained STI diagnosis and treatment service in their life.

#### Substance use

Using addictive substances such as alcohol, ‘khat’ or cigarette with either frequency of; more repeated than daily, daily, weekly or monthly in the past 12 months prior to the study.

#### VCT service utilization

Voluntary Counseling and Testing for HIV is defined as an HIV intervention that includes both voluntary pre and post-test counselling and voluntary HIV testing. In this study, adolescent who ever received HIV counseling and testing service in their life time.

#### Modern contraceptive

Modern contraceptive method is defined as a product or medical procedure that interferes with reproduction from acts of sexual intercourse. These methods are; sterilization (male and female), Intrauterine device and systems, subdermal implants, oral contraceptives, condoms (male and female), injectables, emergency contraceptive pills, patches, diaphragms and cervical caps, spermicidal agents in different form, vaginal rings and sponge. In this study, modern contraceptive usage among adolescents was assessed for intrauterine device, oral contraceptives, condoms (male and female), injectables, emergency contraceptive pills and spermicidal agents.

## Results

### Socio demographic characteristics of the study participants

From the total 682 participants, 648 of them responded to the questionnaires yielded a response rate of 95.01%. Out of 648 participants 348 (53.7%) were males. The mean and median age of the respondents were 17.13 (SD + 1.33), and 17.0 respectively. Six hundred four (93.2%) respondents were Orthodox Christian followed by Muslim 27 (4.2%), and protestant 17 (2.6%). Based on their ethnic group, Amhara ethnic constituted 611 (94.3%), and majority of the respondents 624 (96.3%) were single.

Regarding to their school status, 606 (93.5%) participants were currently enrolled at schools. In contrast, only 5 (0.8%) of the respondents were found without any formal education experience. Among the participants who were attending education, 443 (68.4%) of the respondents reported their educational status as secondary education (Grade 9–12) followed by primary education (Grade 1–8), and above secondary education 132 (20.4%) and 68 (10.5%) respectively.

Concerning the Participants’ mother educational status, nearly similar figures were reported that lies between 20th and 30th percentile with slight percentage increment in mothers with no formal education 175 (27.0%). Out of the total respondents, 412 (63.6%) reported about the absence of discussion habit on sexual and reproductive issues in their family. On the other hand, the median perceived monthly family income was 2095.0 Ethiopian Birr. More than three-fourth, 493 (76.1%) of the study participants were living with their mother and father together (Table 1).

**Table 1:** Socio-demographic characteristics of adolescents of age 15–19 in Debre Berhan town

Variable	Frequency *n = 648*	Percentage (%)
Sex		
Male	348	53.7
Female	300	46.3
Age		
15–17	356	54.9
18–19	292	45.1
Ethnicity		
Amhara	611	94.3
Oromo	15	2.3
Tigre	12	1.9
Gurage	9	1.4
Other	1	0.15
Marital status		
Single	624	96.3
Married	19	2.9
Divorced	2	0.3
Separated	3	0.5
Current enrollment at school		
In school	606	93.5
Out school	42	6.5
Educational status		
No formal education	5	0.8
Primary education	132	20.4
Secondary education	443	68.4
Above secondary education	68	10.5
Mother’s educational status		
No formal education	175	27.0
Primary education	171	26.4
Secondary education	155	23.9
Above secondary education	147	22.7
Habit of communication within family on SRH issues		
Yes	236	36.4
No	412	63.6
Co-residence with both parents		
Yes	493	76.1
No	155	23.9
Perceived monthly income of the family (ETB)		
150–650	35	5.4
651–1400	134	20.7
1401–2350	167	25.8
2351–3550	139	21.5
3551–5000	104	16.0
> 5001	69	10.6

### Respondents’ individual attribute related with sexuality and reproductive health

To assess their knowledge, the study participants were asked about the components of RHS and service delivery points which have a total score of 15. Accordingly, respondents who had high knowledge (scoring above the mean = 8.6 (SD+ 3.4)) were 356 (54.9%). Out of the total participants, 232 (35.8%) have had boy/girlfriends, and among those who had boy/girlfriend, nearly half of them 114 (49.1%) had one boy/girlfriend (mean = 2.06, SD + 1.44). Moreover, one hundred sixty-two (25.0%) of the respondents have had sexual intercourse in their life.

One hundred eighty (27.8%) of the study participants reported that they have had discussion on at least two of the sexual and reproductive health issues [SRH discussion points; Abstinence, condom use, STI/HIV/AIDS, unwanted pregnancy, contraception] with their peers in the last 12 months prior to the study. Discussion on the same issues was also reported by 88 (13.6%), and 82 (12.7%) of the respondents with their sexual partner and with health workers respectively.

Depending on their risky behavior that leads to being infected with HIV virus, the study participants’ perception towards being infected of HIV virus reveals that 559 (86.3%) respondents perceived themselves as free from HIV infection risks.

Regarding to the participants’ last 12-months experience of alcohol, ‘khat’ or cigarette use: majority of them 568 (87.2%) reported that they didn’t consume any of these addictive substances. The remaining use at least one of those drugs at a different regularity. Majority, 28 (33.7%) of the ‘drug-users’ who used those drugs less frequent than monthly followed by weekly 20 (24.1%), monthly 17 (20.5%), daily 15 (18.1%) and more frequent than daily 3 (3.61%). Considering the operational definition used in this study, 55 (8.5%) of the respondents had use those substances (Table 2).

**Table 2:** Adolescents’ individual attribute related with sexuality and reproductive health

Variable		Frequency *n = 648*	Percentage (%)
Knowledge about reproductive health service	High	356	54.9
	Low	292	45.1
Ever had sexual intercourse	Yes	162	25.0
	No	486	75.0
Discuss about SRH issues^a^ with sexual partner in the past 12 months (*n* = 162)	Yes	88	54.3
	No	74	45.7
Discuss about SRH issues^a^ with peer in the past 12 months	Yes	180	27.8
	No	468	72.2
Discuss about SRH issues^a^ with Health worker in the past 12 months	Yes	82	12.7
	No	566	87.3
Perception of risk towards HIV/AIDS	Yes	89	13.7
	No	559	86.3
Exposure to mass-media on SRH issues^a^ in the past 12 months	Yes	484	74.7
	No	164	25.3
Substance use in the past 6 months	Yes	55	8.5
	No	593	91.5

‘^a^’ SRH issues: Abstinence, condom use, STI/HIV/AIDS, unwanted pregnancy, contraception

### Geographical accessibility of reproductive health service delivery points

Regarding to the accessibility of reproductive health service delivery points, Hospitals and Health centers were among the commonly mentioned service delivery points 250 (38.6%) and 239 (36.9%) respectively followed by Clinics (both private and NGOs’) 62 (9.6%). In addition, Pharmacy constitutes 42 (6.5%), Health posts comprise 22 (3.4%), and Youth friendly health service clubs constitutes 17 (2.6%), while Health extension workers comprise 16 (2.5%).

The mean distance from the respondents’ home to the nearest service delivery point was 1.43 km and it was reported to take an average of 19 min to walk. The majority of the participants said that there was RH service delivery center within a 30-min walk (69.4%) and within 1.6 km radius (88.4%) from their home which yields an overall high geographical accessibility of 68.5% and low geographical accessibility of 31.5%. However, there was a slight variation of geographical accessibility between male and female respondents (Table 3).

**Table 3:** Comparison of geographical accessibility and service utilization between male and female adolescents

		Sex		Total, n (%)
		Male (%)	Female (%)	
Distance from home	< 1.6 km	250 (71.8)	200 (66.7)	450 (69.4)
	> 1.6 km	98 (28.2)	100 (33.3)	198 (30.6)
Walking time	Less than 30 min	306 (87.9)	267 (89)	573 (88.4)
	More than 30 min	42 (12.1)	33 (11)	75 (11.6)
Overall Geographical accessibility	High	246 (70.7)	198 (66.0)	444 (68.5)
	Low	102 (29.3)	102 (34.0)	204 (31.5)

### Pattern of reproductive health services utilization among adolescents

**Fig. 1:**
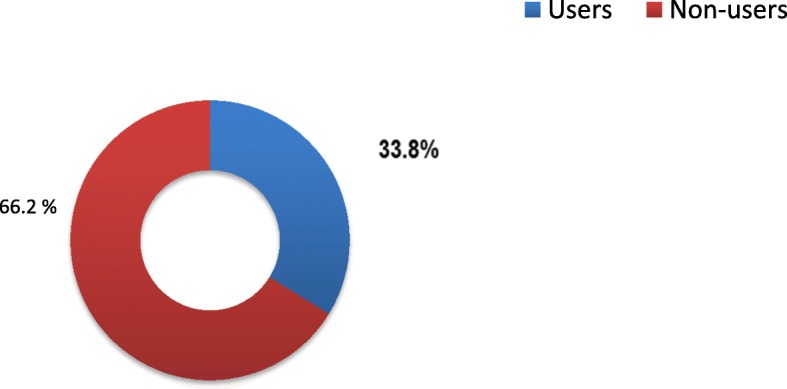
Magnitude of reproductive health services utilization among adolescents

**Fig. 2:**
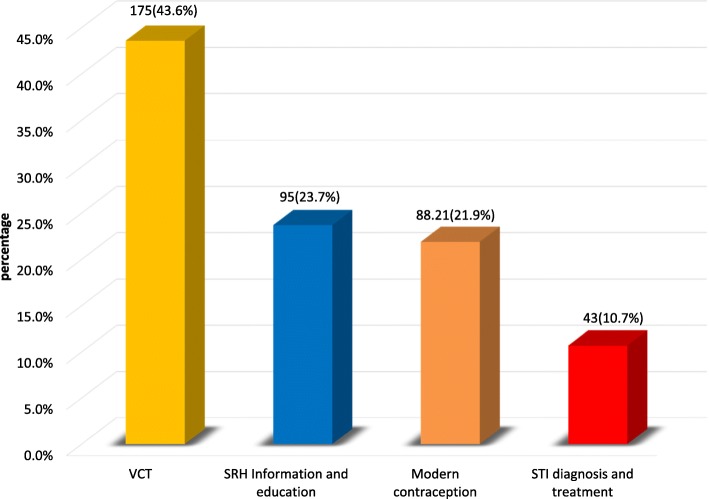
Type of reproductive Health services utilized by adolescents

Nearly, 1 among 3 adolescents, 219 (33.8%) had used at least one of the reproductive health services (see Fig. 1). Accordingly, among these RH service users (*n* = 219), voluntary counseling and testing service for HIV service accounts 175 (43.6%) followed by SRH information and education service users 95 (23.7%), modern contraceptive users 88 (21.9%), and STI diagnosis and treatment service users 43 (10.7%) (See Fig. 2).

**Fig. 3:**
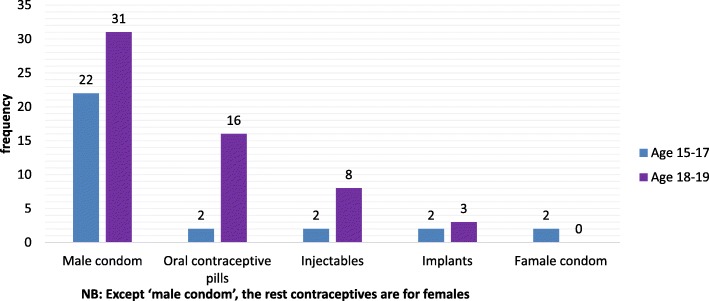
A comparison of modern contraception types used with the age category of adolescents

According to the data obtained on contractive methods used with reference to gender, the most frequently used modern contraceptive method by male adolescents was condom (60.2%) followed by female adolescents using oral contraceptive pills (20.4%) (See Fig. 3).

### Factors associated with RH service utilization

Based on the Bivariate analysis, the factors found to be significantly associated with reproductive health service utilization were adolescents age, marital status, and educational status; mothers’ educational status; poor family communication habit on SRH issues; co-residence with both parents; family monthly income; history of sexual intercourse; poor discussion on SRH matters with sexual partner, pears, and health workers; perception of risk towards HIV/AIDS; exposure towards mass media; addictive substance or drugs use; and geographical accessibility.

With an intention of controlling confounding effect, variables that were statistically significant with reproductive health service utilization on bivariate analysis were interred in to multiple variable logistic regressions. Based on this analysis, adolescents who had discussed sexual and reproductive health issues with their sexual partner and peers were two times more likely to use at least one of the four RH services than their counter parts (AOR = 2.368, 95% CI: 1.168–4.802 and AOR = 2.360, 95% CI: 1.155–4.820 respectively). Adolescents who weren’t co-resided with both their parents were about two times more likely to utilize RH service than those who were living together (AOR = 2.570, 95% CI = 1.155–4.820). Positive perception of oneself towards being infected of HIV/AIDS was a driven force for the adolescents to use RH services twice than those who didn’t perceive themselves as risky (AOR = 2.231, 95% CI: 1.001–4.975) (Table 4).

**Table 4:** Multiple variable logistic regression of factors associated with utilization of RH service

Variables	RHS utilization		COR	AOR
	YES	NO		
Age				
15–17	96	260	1	1
18–19	123	169	1.97 (1.42–2.74)	1.214 (0.56–2.62)
Educational status				
No formal education	2	3	1.36 (0.22–8.21)	1.20 (0.82–6.22)
Primary education (1–8)	39	93	0.85 (0.56–1.30)	1.00 (0.34–2.95)
Above secondary education	32	36	1.81 (1.08–3.03)	1.20 (0.36–3.99)
Secondary education (9–12)	146	297	1	1
Mother’s educational status				
No formal education	66	109	1	1
Primary education (1–8)	73	98	1.23 (0.80–1.89)	0.68 (0.23–2.05)
Secondary education (9–12)	42	113	0.61 (0.38–0.98)	0.68 (0.21–2.15)
Above secondary education	38	109	0.58 (0.36–0.93)	1.13 (0.29–4.39)
Habit of communication with in family on SRH issues				
Yes	106	130	2.16 (1.54–3.02)	1.66 (0.81–3.40)
No	113	299	1	1
Co-residence with both parents				
Yes	148	345	1	1
No	71	84	1.97 (1.36–2.85)	**2.57 (1.07–6.17)** ^a^
Ever had sexual intercourse				
Yes	112	50	7.93 (5.34–11.79)	2.02 (0.89–6.78)
No	107	379	1	1
Perceived monthly family income				
1401–2350	56	111	1	1
150–650	18	17	2.20 (1.01–4.38)	1.80 (0.25–12.80)
651–1400	57	77	1.47 (0.91–2.35)	2.06 (0.71–5.96)
2351–3550	33	106	0.62 (0.37–1.02)	1.19 (0.39–3.69)
3551–5000	32	72	0.881 (0.52–1.49)	1.48 (0.45–4.86)
> 5000	23	46	0.991 (0.55–1.80)	3.04 (0.62–14.98)
Discussion with sexual partner(n = 162)				
Yes	68	20	2.32 (1.17–4.58)	**2.37 (1.17–4.80)** ^a^
No	44	30	1	1
Discussion with peers				
Yes	94	86	2.99 (2.01–4.28)	**2.36 (1.16–4.82)** ^**a**^
No	125	343	1	1
Discussion with health workers				
Yes	47	35	3.08 (1.92–4.94)	2.89 (0.03–8.03)
No	172	394	1	1
Perception of risk towards HIV/AIDS				
Yes	53	36	3.49 (2.20–5.52)	**2.23 (1.00–4.98)** ^a^
No	166	393	1	1
Exposure to mass-media				
Yes	183	301	2.16 (1.43–3.27)	0.80 (0.27–2.38)
No	36	128	1	1
Substance use				
Yes	33	22	3.28 (1.86–5.79)	1.90 (0.73–4.98)
No	186	407	1	1
Geographical accessibility				
High	136	308	0.64 (0.46–0.91)	1.04 (0.45–2.40)
Low	83	121	1	1

^a^statistically significant association

## Discussion

This study revealed that the overall service utilization was 33.8% which is much smaller than study conducted in Mandalay city, Myanmar (67%), and Gondar town, Ethiopia (79.5%) [11, 12]. This variation was possibly due to economical, and socio demographical variations, for example, the study in Myanmar was conducted in the age group of 15–24, and maternal care also included in the service package which may inflate the figure in Mandalay city. In contrast, the study done in Gondar town specifically focused on those adolescents who had sexual exposure which potentially increase the likelihood of service usage. However, adolescents’ service utilization in this study was slightly closer to similar study conducted in Jimma town, Ethiopia which is 41.1% [13].

Succinctly speaking, Twenty-three percent of the participants in this study utilize information and education related with sexual and reproductive health from health care workers, and this finding has concordance with the study done in Jimma town which is 28.8%. With regard to modern contraception utilization, the record in this study which was 21.9% reveals similarity with the results of large scale survey done in 35 countries in Africa on contraceptive methods usage among adolescents of age 15–19 [14]. Family planning service usage in rural areas of East Gojjam and in Jimma town were also 27.7 and 16.3% respectively [13, 15] which didn’t show a great deal of variation with this study findings.

This study revealed that voluntary counseling and testing service utilization for HIV is 43.6%, which has remarkable variation with research findings in Gondar and Goba town 72.2 and 67.3% respectively. This variation could be due to adolescents’ perception of the risk towards HIV virus which was lesser in this study and the recent nationwide noticeable emphasis decrement on the fight against HIV virus related health promotion and prevention activities.

Communication of sexual and reproductive health issues within the family (especially if the family members have good knowledge on reproductive health problems and reproductive health services) allows the adolescent to enhance their knowledge, build their confidence and in turn scale up their tendency to use those services in demand. Unfortunately, 63.6% of the study participants in this study reported that the absence of communication habit on sexual and reproductive health matters within their family. Nearly similar record was obtained from research conducted in East Gojjam area (68.0%) and Goba town (54.2%) [15, 16].

It is obvious that measuring service accessibility needs multiple considerations, but to utilize the services, adolescents’ level of knowledge has an indispensable role. Advocating and increasing awareness about SRH is also central for the success of any adolescent reproductive health program. Knowing which type of service provider can provide the different kinds of services enables adolescents to use services effectively as they needed them. In this study, about 55% of the adolescents had high level of knowledge on RH services and providers compared to 67% in East Gojjam research findings. This could be due to the difference in knowledge assessing tool. The mean score of knowledge assessing questions in this study (mean = 8.6 (SD + 3.4)) exceed two times more than a study that used the same knowledge assessing tool in Mandalay city, Myanmar (mean = 3.3 (SD + 1.6)) [11].

In this study, the researcher found that family factors played an important role in youths’ utilization of RH services. For instance, adolescents who weren’t co-resided with both their parents were 2.57 times more likely to use RH services than those who live together with their parents. This finding is against the study in Gondar town where adolescents living with both of their parents together use VCT service more likely than their counter parts. This could be due to relatively high parental monitoring in our setting. In addition to this, even though majority of the respondents in this study were living with both their parents, the families’ habit of communication on sexual and reproductive issues were quite low (36.2%).

Adolescents’ high-risk perception towards HIV/AIDS drives them to seek and utilize RH service more likely than those who didn’t perceive it. This finding was similar to other studies conducted in Madawalabu University and Gondar town [12, 17]. The above two studies mentioned and other research findings revealed that discussion on sexual and reproductive health matters with sexual partner and peers increase the chance of service utilization [13, 16]. Similarly, this study revealed that adolescents who had discussions on SRH issues with their sexual partners and peers were found to be two times more likely to use SRH services than those who didn’t have the opportunity to discuss the matter.

## Conclusion and recommendations

The findings of this study suggest that RH services among adolescents in the study area were low (33.8%). Voluntary counseling and testing for HIV was the most frequently used service and on the contrary, STI diagnosis and treatment was the low. Co-residence with both parents, discussion with sexual partner and peers, risk perception of oneself towards being infected with HIV/AIDS was among the factors associated with use of RH services.

Therefore, considering the bio-physical and psychological changes of adolescents’ the discussion on sexual and reproductive health matters is highly important and expected from every family. Since majority of adolescents at this age invest most of their time at school, schools can be an excellent means to advance adolescents’ knowledge, attitude and exposure towards RH services. Hence, the school’s administration should endeavor to create conducive environment that helps the adolescents to discuss on their issues and experiences related to RH services. The Regional Health Bureau and North Shoa Zone Health office still needs to reform the existing practice, and establish new comprehensive free standing or integrated adolescent friendly-services with trained service provider in a convenient manner because attempting to achieve prolific outcome require considering adolescents’ need which is dynamic, confidential and flexible by its nature.

## Additional file

Additional file 1: Questionnaire to assess reproductive health service utilization and its associated factors among adolescents in Debre Berhan Town, Central Ethiopia. (PDF 212 kb)

## Abbreviations

EDHS: Ethiopian Demographic and Health Survey
ICPD: International Conference on Population Development
IRB: Institutional Review Board
Kebele*: The smallest administrative structural unit of the town (*local name)
RH: Reproductive Health
RHS: Reproductive Health Service
STD: Sexually Transmitted Disease
VCT: Voluntary Counseling and Testing (for HIV)
WHO: World Health Organization
