# Supplementary material for: Reproductive health services utilization and its associated factors among adolescents in Debre Berhan town, Central Ethiopia: a community-based cross-sectional study
Source: Reprod Health. 2018 Dec 27;15:217. doi: 10.1186/s12978-018-0659-4 (PMC6307180; doi:10.1186/s12978-018-0659-4)
Supplement: Supplementary file 1 — Questionnaire to assess reproductive health service utilization and its associated factors among adolescents in Debre Berhan Town, Central Ethiopia. (PDF 212 kb) [file 12978_2018_659_MOESM1_ESM.pdf]

Title: Questionnaire to assess reproductive health service utilization and its associated factors among adolescents in Debre Berhan Town, Central Ethiopia:

Information sheet

***Title of Research*:** Reproductive health service utilization and associated factors among adolescents age 15-19 in Debre Birhan Town, Ethiopia, 2016.

***Institution*:** Addis Ababa University, College of Health Sciences, School of Allied Health Sciences, Department of Nursing and Midwifery (Graduate Program)

Name of sponsor: Addis Ababa University

***Investigators*:** Kenean Getaneh Tlaye(Msc)

Mesfin Abebe Belete(MSc)

Tefera mulugeta Demelew (MSc)

Mikiyas Amare Getu (MSc)

Feleke Hailemichael Astawesegn (MPH)

***Background Information*:** High level utilization of reproductive health services is an important component in preventing adolescents from different sexual and reproductive health problems. Local evidence about adolescents' reproductive health knowledge, services utilization and associated factors are relevant to design age-appropriate programs, corrective interventions and strategies. Therefore, this study is aimed to investigate the level of reproductive health services utilization and associated factors among adolescents who live in Debre Birhan town, Ethiopia. The study will be conducted in 682 adolescents from 5 randomly selected kebeles by administering pre-tested structured questionnaire.

GENERAL INSTRUCTIONS: The questionnaire has four parts, including questions regarding to socio-demographic and socio-economic characteristics, individual's trends related with sexual and reproductive health, service accessibility and reproductive health service utilization pattern. Please read the instructions and questions carefully before proceeding to answering them.

Part I: <u>Questions assessing the Socio-demographic</u> <u>and</u> <u>socio-economic characteristics of respondents</u>

Instructions: Please circle the number in front of the option you choose. If you are asked to write a response or if your answer is not listed among alternatives, please do in the blank space provided. If there is an arrow in front of your choice, skip to the indicated question.

Q. no.	Questions	Responses	Skip to
101	What is your gender?	1. Male<br>2. female	
102	How old are you?	____________	
103	What is the religion you follow?	1. Orthodox Christian<br>2. Muslim<br>3. Protestant<br>4. Other specify ____________	
104	What is your ethnic group?	1. Amhara<br>2. Oromo<br>3. Tigre<br>4. Gurage<br>5. Other specify____________	
105	Marital status?	1. Single<br>2. Married<br>3. Divorced<br>4. Separated	
106	Are you currently enrolled at school?	1. Yes<br>2. No	

107	In which group of schooling that your educational status categorized?	1. No formal education at all<br>2. Primary education (1-8)<br>3. Secondary education (9-12)<br>4. Above secondary education	
108	In which group of schooling that your mother's educational status categorized?	1. No formal education at all<br>2. Primary education (1-8)<br>3. Secondary education (9-12)<br>4. Above secondary education	
109	Now I have some questions about your family. Does your family have a habit of discussion on sexual and reproductive health (RH) issues with you?	1. Yes<br>2. No	
110	Are you usually living with your mother and father together?	1. Yes →<br>2. No	Q. no 112
111	If No, with whom do you usually live?	1. With my mother only<br>2. With my father only<br>3. With relatives<br>4. With friends<br>5. Alone<br>6. Other specify ____________	
112	How much income do you think your family gain per month? [specify in Birr]	____________________	

Part II: <u>Questions assessing personal characteristics regarding to sexual and reproductive health</u>

Instructions: Here below are some questions regarding to your personal experience regarding to sexual and reproductive health. Please circle the number in front of the option you choose. If you are asked to write a response or if your answer is not listed among alternatives, please do in the blank space provided. If there is an arrow in front of your choice, skip to the indicated question.

Q. no.	Questions	Responses	Skip to
201	Please put '√' mark inside the 'YES' box if you knew the indicated Reproductive Health services and service delivery point (provider) and mark 'NO' otherwise.	**<u>Service</u>**<br>1. Sexual health education and prevention information for young people, single adults, and couples, where confidentiality and privacy are assured.<br>YES ☐ NO ☐<br>2. Sexuality counseling for the client's sexual health concerns or needs, and desired sexuality, reproductive or contraceptive preferences.<br>YES ☐ NO ☐<br>3. Identification and referral for victims of sexual and other forms of violence.<br>YES ☐ NO ☐<br>4. Voluntary counseling, testing, treatment and follow-up for STIs, including HIV.<br>YES ☐ NO ☐<br>5. Diagnosis, screening, treatment and follow-up for reproductive cancers, and associated infertility.<br>YES ☐ NO ☐<br>6. Antenatal, intra-natal and post-natal care for the pregnant women.<br>YES ☐ NO ☐	

		7. Safe abortion to the full extent of the law.<br>YES ☐ NO ☐<br>8. Post-abortion care, including provision of contraceptive information, counseling and methods.<br>YES ☐ NO ☐<br>**<u>Service providers</u>**<br>1. Hospitals. YES ☐ NO ☐<br>2. Health centers. YES ☐ NO ☐<br>3. Clinics (NGO and private).<br>YES ☐ NO ☐<br>4. Health posts. YES ☐ NO ☐<br>5. Pharmacies.<br>YES ☐ NO ☐<br>6. Youth friendly health service clubs.<br>YES ☐ NO ☐<br>7. Health extension workers.<br>YES ☐ NO ☐	
202	Have you ever had a girl/boyfriend? [By girl/boy friend, I mean someone to whom you were sexually or emotionally attracted and whom you 'dated' (use local terms to specify going out together unaccompanied by other person)]	1. Yes<br>2. No →	Q. no. 204
203	How many girl/boyfriend have you had?	____________________	

204	Have you ever had sexual intercourse? [not only with girl/boyfriend]	1. Yes<br>2. No →	Q. no.<br>206
205	From the below listed SRH issues, did you discuss on at least two issues with your sexual partner within the past 12 months? [SRH issues: Condom, STI/HIV/AIDS, Abstinence, unwanted pregnancy, Contraception]	1. Yes<br>2. No	
206	Did you discuss on at least two of the above mentioned issues with your peer within the past 12 months?	1. Yes<br>2. No	
207	Did you discuss on at least two of the above mentioned issues with your health workers within the past 12 months?	1. Yes<br>2. No	
208	Based on prior risky behaviors, Do you perceive yourself as risk for acquiring HIV/AIDS?	1. Yes<br>2. No	
209	Did you exposed to mass media, which aimed to deliver information and education on at least two SRH issues listed below, within the past 12 months? [SRH issues: Condom,	1. Yes<br>2. No →	Q. no<br>301

	STI/HIV/AIDS, Abstinence, unwanted pregnancy, Contraception]		
210	Which mass media did you exposed for?	1. Radio<br>2. Television<br>3. Magazine<br>4. Newspaper<br>5. Pamphlet<br>6. Other specify________________	
211	Did you use alcohol, khat or cigarette in the past 12 months?	1. Yes<br>2. No ——————————→	Q. no<br>301
212	If YES, to what frequency?	1. More frequent than daily<br>2. Daily<br>3. Weekly<br>4. Monthly<br>5. Less frequent than monthly<br>6. Other specified ____________	

Part III: <u>Questions assessing service accessibility</u>

Instructions: Dear respondent, afterwards there are some questions regarding to accessibility of RH service delivery points. Please circle the number in front of the option you choose. If you are asked to write a response or if your answer is not listed among alternatives, please do in the blank space provided.

Q. no.	Questions	Responses	Skip to
301	What is the nearby RH service delivery point (service provider) to your home?	1. Hospitals<br>2. Health centers<br>3. Clinics (NGO and private)<br>4. Health posts<br>5. Youth friendly health service clubs<br>6. Health extension worker<br>7. Other specify___________	
302	How far the above mentioned nearby RH service delivery point from your home? [specify in km]	___________________	
303	How much it takes to walk from your home to the nearby RH service delivery point? [specify in minute]	____________________	

Part IV: Questions assessing Reproductive Health service utilization

Instructions: Questions below are regarding to your service usage status. Please circle the number in front of the option you choose. If you are asked to write a response or if your answer is not listed among alternatives, please do in the blank space provided. If there is an arrow in front of your choice, skip to the indicated question.

Q. no.	Questions	Responses	Skip to
401	Have you ever use any RH service in life?	1. Yes<br>2. No →	**You are finished here, thank you!**
402	Have you received any information and education service regarding to SRH issues from health worker working in any of RH service delivery points in the past 12 months?	1. Yes<br>2. No →	Q. no 404
403	If YES, what type of information and education you received?<br>[you can encircle more than one response]	1. Information and education related to sexual health<br>2. Information and education related to Contraception<br>3. Information and education related to SIT diagnosis, and treatment<br>4. Information and education related to VCT<br>5. Information and education related to safe abortion	

		6. Information and education related to Antenatal, Intranatal and Postnatal care<br>7. Other specify_________	
404	Have you use modern contraceptive service in the last 12 months?	1. Yes<br>2. No →	Q. no 406
405	If YES, what type of method do you use? [methods other than male condom are filled by female respondents' only]	1. Male condom<br>2. Pill<br>3. Injectable<br>4. Implant<br>5. IUCD<br>6. Female condom<br>7. Other specify _______	
406	Have you ever used VCT service?	1. Yes<br>2. No	
407	Have you ever used STI diagnosis and treatment service?	1. Yes<br>2. No	

THANK YOU!
